# Sex Workers’ Lived Experiences With COVID-19 on Social Media: Content Analysis of Twitter Posts

**DOI:** 10.2196/36268

**Published:** 2022-07-14

**Authors:** Ahmed Al-Rawi, Kiana Zemenchik

**Affiliations:** 1 School of Communication Simon Fraser University Burnaby, BC Canada

**Keywords:** sex work, social media, COVID-19, pandemic, Twitter, infodemiology, social stigma, sex worker, risk, public health

## Abstract

**Background:**

The COVID-19 pandemic has drawn attention to various inequalities in global societies, highlighting discrepancies in terms of safety, accessibility, and overall health. In particular, sex workers are disproportionately at risk due to the nature of their work and the social stigma that comes alongside it.

**Objective:**

This study examines how public social media can be used as a tool of professional and personal expression by sex workers during the COVID-19 pandemic. We aimed to explore an underresearched topic by focusing on sex workers’ experiences with the ongoing COVID-19 pandemic on the social media platform Twitter. In particular, we aimed to find the main issues that sex workers discuss on social media in relation to the COVID-19 pandemic.

**Methods:**

A literature review followed by a qualitative analysis of 1458 (re)tweets from 22 sex worker Twitter accounts was used for this study. The tweets were qualitatively coded by theme through the use of intercoder reliability. Empirical, experimental, and observational studies were included in this review to provide context and support for our findings.

**Results:**

In total, 5 major categories were identified as a result of the content analysis used for this study: concerns (n=542, 37.2%), solicitation (n=336, 23.0%), herd mentality (n=231, 15.8%), humor (n=190, 13.0%), and blame (n=146, 10.0%). The concerns category was the most prominent category, which could be due to its multifaceted nature of including individual concerns, health issues, concerns for essential workers and businesses, as well as concerns about inequalities or intersectionality. When using gender as a control factor, the majority of the results were not noteworthy, save for the blame category, in which sexual and gender minorities (SGMs) were more likely to post content.

**Conclusions:**

Though there has been an increase in the literature related to the experiences of sex workers, this paper recommends that future studies could benefit from further examining these 5 major categories through mixed methods research. Examining this phenomenon could recognize the challenges unique to this working community during the COVID-19 pandemic and potentially reduce the widespread stigma associated with sex work in general.

## Introduction

### Background

There is some ethnographic research discussing the relationship between the COVID-19 pandemic and female sex workers’ experience as a marginalized group in society, but there is a clear lack of literature discussing how public social media can be used as a tool of professional and personal expression amongst sex workers. We focus here on public social media in contrast to closed or restricted social media sites, such as OnlyFans. Though sex workers are a vulnerable group, social media provides people with vocal autonomy and advocacy, while also finding a sense of belonging and community [1]. Thus, social media is an important tool for drawing attention to some of the major concerns and issues experienced by sex workers during the COVID-19 pandemic. Though there are studies that have focused on the challenges experienced by sex workers more generally, there is a lack of literature examining the ways that sex workers use social media.

This gap in academia reinforces the argument that sex workers are both vulnerable and devalued and fails to acknowledge how social media can act as an important tool for developing an online community, encouraging safety, and developing a dialogue between vulnerable groups, such as sex workers, and the public. It is important to note that most of the literature focuses on female sex workers, but some studies have examined the implications and stigma associated with male sex workers [2-4]. Regardless of the gender of participants, it is undeniable that the stigma associated with sex work reinforces the vulnerability of this group before the pandemic and continues to render them even more vulnerable today.

Despite the ground that existing research has covered, there is still a lack of studies exploring how sex workers use social media to amplify their voices, discuss their personal experiences, and demonstrate support for other vulnerable groups. As a result, this study aims at filling some of these gaps by exploring how sex workers use social media to disseminate information about COVID-19 and their experiences while working during the pandemic.

### Literature Review

In general, existing academic studies concerning sex workers’ use of social media platforms have not fully explored the implications of social media usage during precarious times, such as during the ongoing COVID-19 pandemic. Although several available studies have examined the implications of sex workers’ use of social media [1,2,4-6], there is a limited number of academic studies that have empirically explored and analyzed existing social media posts from sex workers themselves to determine how social media platforms function as important sites for self-identified sex workers in a multitude of ways during the COVID-19 pandemic.

The available studies highlight the importance of ensuring that sex workers have adequate access to health care resources, particularly during the COVID-19 pandemic. Indeed, almost all types of sex work remain highly stigmatized [7], which has led to the creation of various organizations that fight for sex workers’ basic rights to ensure that their positions are seen not only as work but also as essential work [1,8]. In fact, some studies and reports concluded with calls of action that specifically identified the discrepancies between work that is, on the one hand, considered essential and, on the other hand, considered sex work, thus drawing attention to the stigma associated with the latter [3,7,9]. Many sex workers are unable to access their health care needs due to government restrictions, long wait times, and limitations on social gatherings [8]. Though the demand for sex work during the pandemic remains strong, sex workers are not granted the same precautions that workers in other industries are, especially with regard to economic and labor support [10], a lack of available and accessible COVID-19 testing [7], and a higher risk for the spread of sexually transmitted diseases [8]. Due to inadequate resources and health care, many sex workers have to rely on their own strategies for their safety and advocacy.

Several published academic studies concerning the implications of COVID-19 and sex workers have been explored worldwide. In Ghana and Kenya, sex work itself is not deemed illegal, though it is not considered essential work [3,11]. The Kenyan and Ghanaian governments have put several regulations in place to prevent the spread of the virus. Though this has reduced the spread of the virus, it has threatened many sex workers’ stability and only added to their vulnerability [3,11]. Through various social media channels, sex workers in Kenya are able to share information with one another and discuss safety strategies [3]. The instability of work for sex workers is not limited to the West; thus, it is a reality for sex workers worldwide that can be somewhat mitigated through the use of social media channels. This highlights that although the nature of sex work has depended on being in close physical contact with others, the integration of social media platforms provides sex workers with alternative and safer options for their line of work.

Several studies have highlighted the various purposes social media usage can serve for sex workers, sex work organizations, and other vulnerable groups. One recent study found that social media can function as a tool for advocacy amongst the sex worker community [1]. In the study, the authors highlighted the many benefits of encouraging sex workers to use social media, as well as the potential risks involved with using these platforms [1]. Though activities that are both online and offline have similar characteristics, it is worth noting that not all digital spaces are accessible to all sex workers, even if there has been an influx of sex workers relying on these online spaces [12,13]. In addition, another study found that social media advocacy can increase the efficiency of moving between offline and online spaces, enhance safety, and strengthen communication between sex workers [1]. In particular, the authors found that sex workers who were engaged in dialogue with one another would have many more positive encounters with clientele, as they could exchange information about potential clients and discuss problems concerning specific clientele [1]. In the past, sex workers have been limited to who they encounter in the streets [4], but now, given the ubiquity of social media, sex workers can instantly connect with one another and transmit information in a much more efficient, safe, and effective manner.

In general, the advent of social media has changed how sex workers engage with and meet their clients, promote their services, and explore their agency online. A different study found that online spaces provide extensive opportunities for sex workers to explore and curate their online identities to collaborate with one another, to avoid conflict, and to develop a stronger role of agency [6]. Although social media has proven to have many benefits for sex workers, its use is not without risks. One study found that sex workers struggle with receiving payment, especially since social media can act as a transparent space where content, such as photos and videos, is already visible on one’s profile for free [4]. Although social media offers many benefits for sex workers, these sites call for a lot more emotional and mental labor to maintain and manage one’s online profile.

One of the challenges not mentioned in these aforementioned studies is that of shadowbanning, which is a phenomenon vital to understanding how sex workers navigate online spaces. Shadowbanning is a process that reduces and limits the visibility of a user’s social media platform, even to their followers [14,15]. Shadowbanning has become a lot more prominent for sex workers on social media platforms with an audio-visual focus, such as Instagram, OnlyFans, TikTok, and Facebook. According to 1 sex worker who identifies as a camgirl who has worked in the industry for over 10 years, most social media platforms frequently discriminate against sex workers’ persons, work, and jobs, rather than their content [14]. This is believed to be in relation to the US Fight Online Sex Trafficking Act–Stop Enabling Sex Traffickers Act (FOSTA-SESTA) bill, which was enacted to prevent online sex trafficking, though sex work is consensual and legal for many sex workers online. This bill has increased social stigma toward sex work as it does not differentiate between consensual and nonconsensual sex work sectors [14]. However, Twitter has proven to be much less extreme in shadowbanning sex workers, whereas Instagram has made it extremely difficult for users to search for sex workers’ accounts through the search function, as well as limiting the visibility of posts and stories of sex workers, even for their followers [15,16]. Thus, the shadowbanning techniques implemented by Twitter appear to be less unrelenting compared to other social media platforms, but the platform is still not completely accepting and inclusive of sex workers.

What is largely missing in these studies is an empirical examination of sex workers’ social media posts referencing the pandemic. Therefore, this study attempts to answer the following main research question: *What are the main issues that sex workers discuss on social media in relation to the COVID-19 pandemic?*

## Methods

### Data Collection

Social media data for this study were collected from December 29, 2019, until July 7, 2021, which is when the study was conducted. The data collection began in late 2019 because this is when coronavirus emerged. Using convenience sampling, 2 researchers examined the Twitter profiles of 22 sex workers, which were identified using several English search terms, such as “sexwork” and “sex workers.” The identified users included 15 (68.2%) women; 5 (22.7%) sexual and gender minorities (SGMs), including 1 (20%) individual who is openly transgender; and 2 (9.1%) men. For this study, SGMs should be understood as those who identify as being part of the two-spirit, lesbian, gay, bisexual, transgender, queer or questioning, intersex, asexual (2SLGBTQIA+) community, those who identify as nonbinary, and those who are not cisgender. Most of the sex workers have self-identified their gender on their Twitter profiles. Interestingly, 9 (40.9%) sex workers on our list publicly declared on their Twitter profiles that they are vaccinated.

In this respect, we cannot claim that we managed to map all the available sex workers on Twitter, as this is a prodigious if not an impossible task, but we believe that the number of users was adequate to conduct this study. We used convenience sampling for this study, where users were selected based on their availability; the chosen profiles have identified that they are sex workers in their descriptions, and their profiles are public, in which their posts are available to all users and viewers. We chose to use Twitter for this study due to the higher prominence of shadowbanning on different social media platforms and its higher level of accessibility for sex workers. Though not without controversy, Twitter acts as a more accessible and less problematic platform for sex workers as it is one of the few that does not censor users solely based on their career, and the guidelines are much easier to navigate [15,17].

### Data Extraction

To extract the data, we used Twitter application programming interface (API) v2, which allows full data extraction, except for deleted posts or private accounts. In total, 134,025 tweets were downloaded, representing all the available messages posted on the accounts of the 22 sex workers, including what they retweeted. We included the retweets in our analysis because they also represent what the sex workers intended to highlight, such as violations or work restrictions. On average, each sex worker posted 6092 (re)tweets during the study period; the highest number of (re)tweets was 31,802 by 1 (4.5%) sex worker, and the lowest was only 3 (re)tweets. Multimedia Appendix 1 shows that March 14, 2021, had the highest number of (re)tweets.

As the collected data were rather substantial for a manual content analysis, we used a Python script [18] to extract tweets that referenced 33 terms related to the pandemic: “coronavirus,” “pandemic,” “coronapocalypse,” “COVID,” “COVID-19,” “COVID19,” “vaccin*,” “vaxxed,” “mRNA,” “Pfizer,” “Moderna,” “Johnson & Johnson,” ““Johnson&Johnson,” “Astra-Zeneca,” “Covidiot,” “essential worker,” “frontline worker,” “herd immunity,” “virus,” “quarantine,” “epidemic,” “asymptomatic,” “personal protective equipment,” “PPE,” “contact tracing,” “lockdown,” “new normal,” “social distancing,” “social distance,” and “N95.” Another Python script was used to extract the most referenced hashtags in the overall data set. Once again, these search terms do not represent all the available terms that are related to the pandemic, but they cover the main issues concerning COVID-19. The filtered data comprised 1458 (re)tweets that received 11,035 likes, 960 replies, and 10,517,689 retweets. To design the codebook, 2 coders examined a sample data set using emergent coding [19,20] to identify the major categories in the social media posts. In total, 6 main categories were identified as follows:

- Blame: Tweets that blame and criticize the government, rich people, and those in positions of power, such as health authorities, for how the virus is being handled, how the vaccine is being distributed, etc.
- Humor: This is generally used as a coping mechanism or to increase people’s ability to relate to what others are feeling. Many people will use the retweet or like function to show that they agree with it or to share these tweets with their followers to make others laugh about some unique circumstances occurring during the pandemic.
- Concerns: Tweets that express health concerns or concerns about their loved ones or people they know, as well as concerns about fake news, media reliability, race, class, and gender—intersectionality—across social groups, including concerns about essential workers, etc.
- Solicitation: Tweets that promote sex workers’ availability, vaccine status, times that they will be online or available for meetings, or cities that they will be traveling to. This category also includes when users post photos of themselves.
- Herd mentality: This category refers to the way people will try to create an emotional plea or rational argument to conform to mainstream ideas and policy guidelines; tweets will normally say “retweet if you’re still social distancing” or “have you taken the vaccine yet?”. This is similar to shaming but is based on being socially desirable by following public health regulations.
- Other: Any other minor issues that are not listed in the previous categories.

To test the codebook, 2 coders examined over 10% (n=150) of the total data set, and in the second attempt after a few rounds of deliberation and discussion, intercoder reliability was measured using Krippendorff α≥0.78, which was acceptable [21].

### Ethical Considerations

As mentioned, sex workers are a highly vulnerable group that is also highly stigmatized. When researching such a population, it should be ensured that the privacy and confidentiality of the participants are protected. Given that the data for this study were collected through users’ public profiles on social media, ethics clearance was not needed from our university. That being said, though these users have their profiles set to “public,” that does not mean that it is any less incriminating if their identities are revealed [22]. To address this ethical concern, this study did not provide any personal details about any of the participants, where names, locations, and specific details from their profiles were all omitted. The method for this study was broad enough that others using similar search terms in the Twitter search bar may not receive the same results—due to algorithms or other factors. To further protect the identities of the participants, our method included both original tweets and retweeted posts, so it is impossible to identify the sex workers examined in this study as the increased number of posts further protects the participants.

## Results

### Main Findings

As mentioned before, this study aims to identify the major themes and topics sex workers discuss on the social media platform Twitter. From the 1458 tweets and retweets, 6 main categories emerged: blame, humor, concerns, solicitation, herd mentality, and other. These categories are exhaustive and mutually exclusive, and the coders identified the most dominant underlying message in each tweet, especially due to the brevity of tweets. To ensure the validity of the codebook, 2 coders examined a sample and the intercoder reliability test using Krippendorff α≥0.78 was acceptable [21].

### Blame

Blaming others is a reactionary retort that can address one’s frustration. For this study, the blame category is understood as posts that explicitly and critically identify an actor responsible for the cause of concern. These actors are criticized and can include government representatives, political parties, the elite, police departments, and others in positions of authority. Although the rationale behind blaming or criticizing others may vary, it can be agreed that blaming others in times of uncertainty is a common reaction, especially if individuals are experiencing a heightened level of fear and anxiety [23,24]. This category came as the fifth-most frequent one, with only 146 (10%) of the social media posts referencing it (Multimedia Appendix 2). Some examples of the blame category include statements resembling criticism, such as:

How ICE helped Spread the Coronavirus...

Rich people did not experience the same pandemic as working class people and now they get the vaccine first. It’s actually twisted.

In terms of gender, we found that SGM sex workers had the highest percentage of tweets that blame and criticize other parties (n=62, 11.2%), followed by female sex workers (n=83, 9.3%), denoting the urgent issues these groups discuss. Twitter audiences did not show active engagement with messages that contained blame, for this category came fifth, with 1,790,953 (17%) of the most retweeted posts.

### Humor

Humor is a powerful coping mechanism that takes on multiple forms on social media and is especially prominent in times of crisis, such as during a global pandemic. With this in mind, humor is understood as the way that users rely on funny posts in their many forms—including sarcasm, satire, and irony—as a coping mechanism and to produce relatable content. Humor is a vital tool for bringing hope to people while they are experiencing times of crisis; humor is also understood as 1 of the only “available option[s]” for openly criticizing others, releasing one’s frustration, and providing a narrative for living through a pandemic [25,26]. This category came in fourth at 13%, with 190 of the social media posts referencing it (Multimedia Appendix 2), and male sex workers (n=4, 28.5%) seem to rely on this category more often than other gender groups (Table 1); however, the data on men are extremely low, so definite conclusions cannot be reached. Some examples of humor in this data set include:

Feel bad for people who got fit in quarantine but now have to STAY fit for like 7 more months before anyone sees. Shoulda paced yourselves

Whoever smoked mid from a soda can as a teenager is immune to coronavirus

Interestingly, social media audiences found this category more appealing than other ones for they mostly retweeted posts in the humor category, at 3,205,669 (30.4%) of the most retweeted posts.

**Table 1:** Major categories (N=6) discussed by sex workers by gender.

Category	Women (N=886), n (%)	Men (N=14), n (%)	SGM^a^ (N=558), n (%)
Blame	83 (9.3)	0	63 (11.2)
Humor	120 (13.5)	4 (28.5)	66 (11.8)
Concerns	302 (34.0)	6 (42.8)	234 (41.9)
Solicitation	221 (24.9)	3 (21.4)	112 (20.0)
Herd mentality	155 (17.4)	0	76 (13.6)
Other	5 (0.5)	1 (7.1)	7 (1.2)

^a^SGM: sexual and gender minority.

### Concerns

The number of concerns that have emerged since the outbreak of coronavirus have been multifaceted and expansive. Thus, for the purposes of research, concerns are understood as being associated with individuals’ worries about their loved ones, health issues, essential workers and businesses, and intersectionality [27]. The latter refers to how the lived experiences of someone’s life can impact one’s “social determinants of health” due to their identity [28]. This category of concerns therefore also refers to those whose identities render them marginal, such as those who are immunocompromised or racialized. Concerns not only refer to fears associated with contracting or spreading coronavirus but also include concerns about public safety, public health, and those who are especially vulnerable to the virus.

The findings showed that this is the top category in the whole data set, constituting 542 (37.2%) of the posts (Multimedia Appendix 2). It is also the highest category across all gender groups (men: n=6, 42.8%; SGMs: n=234, 41.9%; and women: n=302, 34.0%; see Table 1). One example is:

in the 8 days since the first COVID-19 case on the navajo nation, our nation now has 49 cases. that number is growing every day. i’m starting a thread of all the ways you can support the navajo nation during the COVID-19 pandemic. ⬇️

Another example states:

The Covid-19 pandemic particularly has demonstrated how so many people who already face marginalisation are excluded from vital financial resources - government schemes, access to bank accounts and cards. Denying people access to these denies them access to society as a whole.

In terms of the most referenced hashtags, we found that sex workers used relevant terms to express different types of legal, safety, and health concerns, such as #covid19 (n=52), which is ranked amongst the top 10 most used hashtags, as well as other ones, such as #sexworkerdemands (n=18); #antitrafficking (n=15); #sextrafficking (n=15); #coronavirus (n=14); #decrimqld (n=12), used in relation to decriminalizing sex work in Queensland, Australia; and #antiprostitution (n=6). Some of the other concerns expressed by sex workers were related to their online safety, for a few of them mentioned the importance of avoiding doxxing, which is the online disclosure of personal information, such as sex workers’ phone numbers and addresses. For example, 1 (4.5%) sex worker retweeted:

Harm Reduction: Doxxing Prevention Tips for Sex Workers and Protesters is now live!

In terms of the audience’s reaction, this category came second in relation to the retweeted posts, comprising 2,787,188 (26.5%) of the retweeted posts.

### Solicitation

The COVID-19 pandemic has presented sex workers with many challenges, which include the way that they promote and solicit their work. Although there were some sex workers who had already moved to online platforms, the accessibility of Twitter provides sex workers with an online platform where they can solicit online clients by promoting their services or performing their gender in a specific way [6,29]. This category of promotion and solicitation, therefore, includes content that promotes the availability of sex workers’ services, such as photographs of the worker in question, information about their vaccine status, cities that they will be traveling to, and specific dates and times that they will be available for meetings. This is an expansive and comprehensive category that not only includes sex workers promoting themselves but could also include the promotion of other organizations that need financial or social support that aligns with sex workers’ individual values. This is the second-most frequent category, with 336 (23%) posts (Multimedia Appendix 2), and female sex workers posted more tweets around this issue (n=221, 24.9%; see Table 1). Some examples of this include:

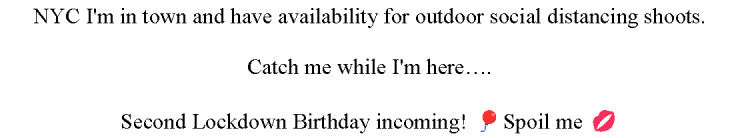



Finally, this category of posts received the least amount of attention from Twitter audiences, with 32,047 (0.3%) retweeted posts.

### Herd Mentality

As mentioned before, herd mentality refers to how the actions and behaviors of a group can influence other individuals. During the COVID-19 pandemic, herd mentality has often been associated with promoting messages that align with government recommendations and public health authorities’ guidelines to reduce the number of cases of COVID-19 among the population. Those on social media will often promote government restrictions, information about how to social distance at protests, or promote getting vaccinated. Although there is no central authority or specified leader to herd mentality, this kind of behavior and its associated actions are transmitted to local networks with the help of others who explicitly promote this way of thinking [30,31]. In this case, the local actions can refer to social media posts, such as in the form of (re)tweets, to promote these messages. This category came third, with 231 (15.8%) posts, as the most important issue discussed by sex workers (Multimedia Appendix 2), and female sex workers are ahead of other groups in referencing it (n=154, 17.4%), followed by SGMs (n=76, 13.6%; see Table 1. Some examples of herd mentality include:

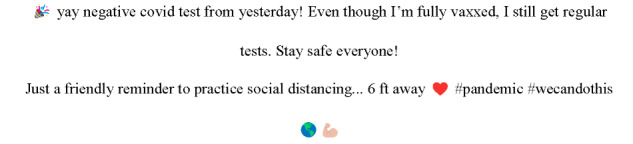



In terms of audience engagement with these types of tweets, this category only came third, with 2,653,060 (25.2%) of the retweeted posts.

Finally, the other category is not discussed here, because it deals with only minor issues that are mostly personal or irrelevant to this study, comprising 13 (0.8%) of the retweeted posts.

## Discussion

### Principal Findings

As described in this paper, most tweets in our sample align with the issue of concerns. The ongoing COVID-19 pandemic has drawn significant attention to the inequitable structures in society that disproportionately impact vulnerable groups. As a result, the pandemic has influenced how sex workers advocate for themselves on social media platforms, as they are more likely to express concerns about how COVID-19 has impacted their own work and other communities worldwide. Although the concerns have been localized to certain national contexts, such as the United States and Australia, the nature of this online community through the digitization of advocacy has highlighted how COVID-19 is a global phenomenon, thus causing sex workers to advocate for themselves and their own work alongside other disparate groups worldwide. For example, we found that the hashtag #blacklivesmatter was mentioned 50 times by sex workers, together with #BLM (n=31) and #blacklivesmatteraustralia (n=19), to express solidarity with the movement. Though this could be due to the large wave of support for the Black Lives Matter movement following the murder of George Floyd, this highlights that sex workers, as a marginalized community, will not only use social media as a site to discuss their own difficulties but also draw attention to how other social groups have been impacted [28]. The same point applies to intersectional issues and politics.

Given that concerns are closely associated with protecting vulnerable populations, we found that herd mentality was the third-most frequent category. The implicit part of addressing concerns about the vulnerability of certain groups is ensuring that one is taking the necessary steps to protect themselves and others from contracting and spreading the virus. By following the official COVID-19 guidelines, sex workers examined in this study are highlighting the importance of following restrictions to keep others safe and, by showing that they are taking part in this behavior, others should too. Since this category was the third-most frequent one, it is closely associated with the most recurrent category of concerns because, to protect those who are more vulnerable, everyone needs to follow the official health guidelines.

Solicitation was the second-most frequent category. Given that COVID-19 is highly contagious and sex work is a profession that often requires close physical contact between parties, sex workers are at a much higher risk for contracting COVID-19. The codebook sample provided insight into how sex workers were struggling to find work during the pandemic, especially since sex work is not decriminalized or legalized in some countries [11]. Thus, the high frequency of tweets that were coded as solicitation demonstrates how sex workers are using social media platforms to promote their services and have an income to rely on.

The category of humor was not only used by sex workers during the COVID-19 pandemic but also relied upon by many individuals as a coping mechanism [25,26]. Through the use of humor, the severity of the pandemic can be momentarily forgotten as users share relatable information in order to make light of the situation. Though humor was the fourth-most frequent category, its presence shows that the sex workers from this sample engaged with these posts during times of crisis, though there was a much stronger presence of other serious themes.

Finally, the blame category was the least frequent, where those in positions of power are criticized or identified as the cause of concern. During a global health crisis, it is only natural that vulnerable populations will point out further issues of inequity with regard to the specific challenges faced by their community (as is the case with the concerns category) or through the identification of a single actor or representative engaging in activities that are a cause for concern. By identifying different actors that are believed to be responsible for social inequalities or, at the very least, perpetrating them, sex workers are drawing specific attention to the root causes of inequity and how it is manifested in the society during the COVID-19 pandemic.

### Limitations

One of the limitations of this study is that we only used posts and retweets that were in the English language. It is likely that there are many other sex workers’ accounts on Twitter that are not written in English, but they were not included in this study. This was not the only factor that limited our scope: While conducting our search, we used a limited number of search terms and only used Twitter, while some sex workers might prefer to use other online platforms, such as OnlyFans and Instagram. That being said, Twitter was the most reliable platform for this study because other social media platforms are a lot stricter with shadowbanning content, thereby making it more difficult to find and identify sex workers on those platforms.

Though the authors believed that they reached saturation with 22 sex workers’ accounts for this study, it is likely not representative of the sex worker community as a whole. There are many unique challenges that sex workers in different countries experience, which may not be summed up in only 280 characters. Furthermore, there are sex workers who may not feel comfortable disclosing their profession on their Twitter account for personal or safety reasons, just as there are likely sex workers who do not use Twitter at all.

### Conclusion

Although the existing literature vaguely highlights how sex workers use public social media as tools of advocacy, education, and community support in this community, this study used a selected sample of tweets to examine the specific issues that arise from the content of tweets on sex workers’ public accounts. The 6 main issues of blame, humor, concerns, solicitation, herd mentality, and other discerned from this sample highlight how Twitter functions as an uncensored space for sex workers to openly discuss their lived experiences while working in an essential, yet high-risk, career throughout the pandemic. These issues, some of which are pressing ones, do not only highlight sex workers’ individual concerns but demonstrate that sex workers are advocating for and supporting other vulnerable communities through retweets and likes and posting their own content about other essential workers and those with marginal identities.

Future research on the topic of sex workers’ online advocacy could benefit from examining these issues in detail through mixed methods research, such as through online surveys, interviews, or focus groups that explicitly seek answers to questions about sex workers’ online advocacy strategies. Examining this phenomenon is not only significant for recognizing the challenges unique to this community during the COVID-19 pandemic but can also highlight how online spaces function as alternative locations of inclusion without the normally ubiquitous stigma attached to sex work. Indeed, social media can be effectively used as a tool for advocacy, education, and community support.

## Appendix

Multimedia Appendix 1 Frequency of tweets posted by 22 sex workers.

Multimedia Appendix 2 Percentages and frequencies of tweets by category.

## Abbreviations

SGM: sexual and gender minority

## Data Availability

The data set and the coded posts are available with the researchers and can be shared with the reviewers upon request. However, we cannot post them online in a public data repository in order to protect the identity of sex workers.
