# Supplementary figures and images for: Sex Workers’ Lived Experiences With COVID-19 on Social Media: Content Analysis of Twitter Posts
Source: JMIR Form Res. 2022 Jul 14;6(7):e36268. doi: 10.2196/36268 (PMC9285671; doi:10.2196/36268)

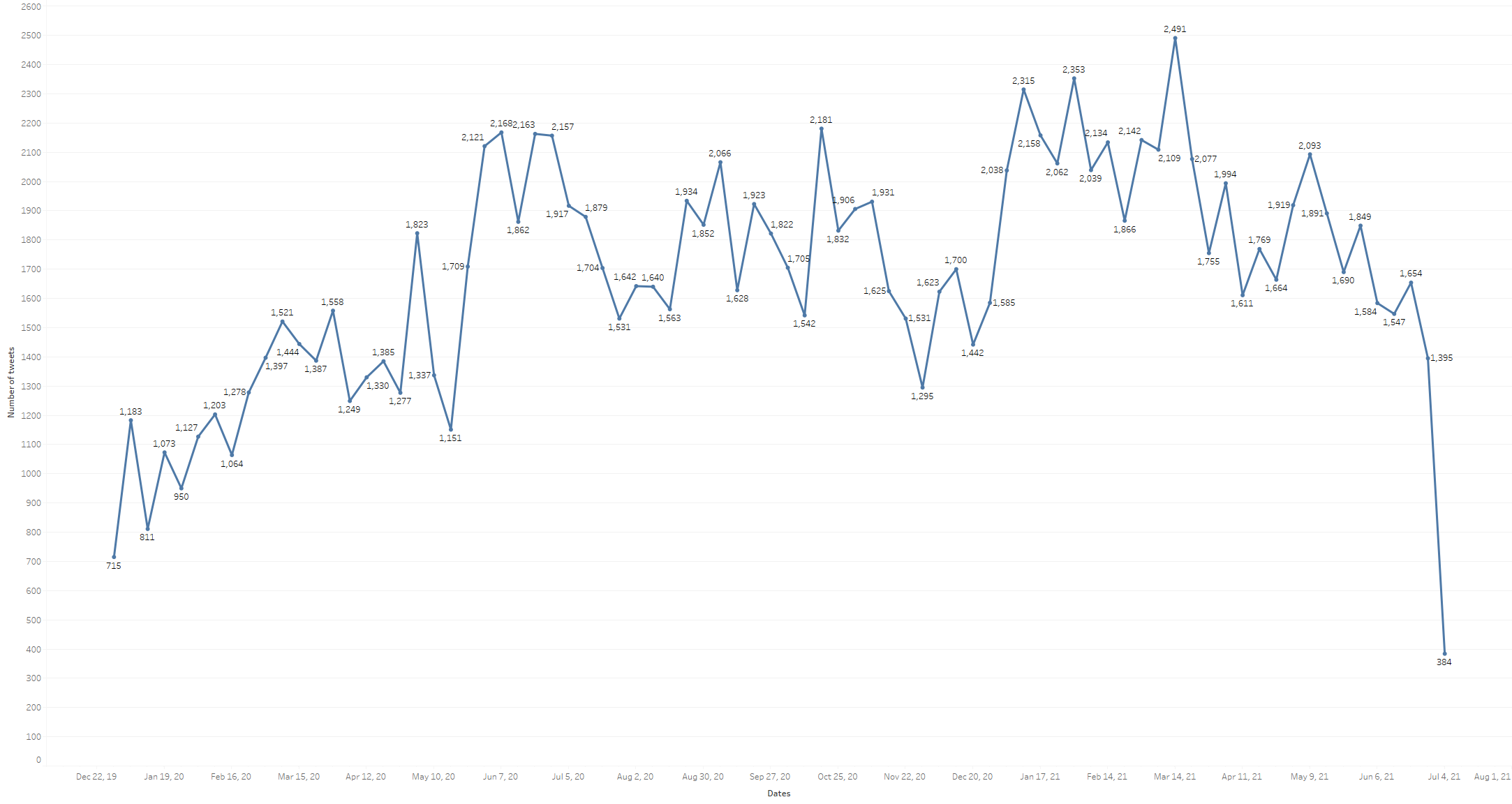

Supplement: Multimedia Appendix 1 [file formative_v6i7e36268_app1.png]

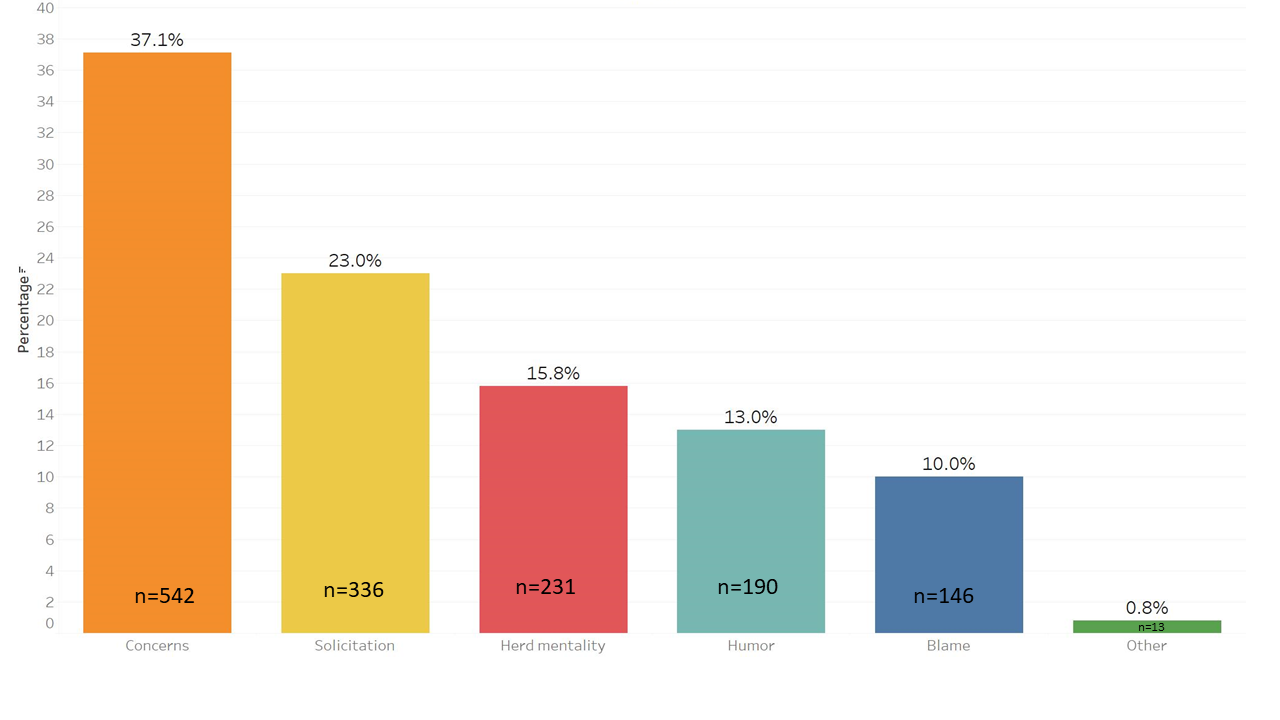

Supplement: Multimedia Appendix 2 [file formative_v6i7e36268_app2.png]
